# Overview of Fiber Optic Sensor Technologies for Strain/Temperature Sensing Applications in Composite Materials

**DOI:** 10.3390/s16010099

**Published:** 2016-01-15

**Authors:** Manjusha Ramakrishnan, Ginu Rajan, Yuliya Semenova, Gerald Farrell

**Affiliations:** 1 Photonics Research Centre, School of Electrical and Electronic Engineering, Dublin Institute of Technology, Kevin Street, Dublin 8, Ireland; yuliya.semenova@dit.ie (Y.S.); gerald.farrell@dit.ie (G.F.); 2 School of Electrical, Computer and Telecommunications Engineering, University of Wollongong, New South Wales 2522, Australia; ginu@uow.edu.au

**Keywords:** fiber optic sensor, composite materials, strain/temperature sensing, smart materials, structural health monitoring

## Abstract

This paper provides an overview of the different types of fiber optic sensors (FOS) that can be used with composite materials and also their compatibility with and suitability for embedding inside a composite material. An overview of the different types of FOS used for strain/temperature sensing in composite materials is presented. Recent trends, and future challenges for FOS technology for condition monitoring in smart composite materials are also discussed. This comprehensive review provides essential information for the smart materials industry in selecting of appropriate types of FOS in accordance with end-user requirements.

## 1. Introduction

Composite material structures [1] are widely used in the aerospace, marine, aviation, transport, sport/leisure and civil engineering industries [1]. Composite material structures are frequently subjected to external perturbations and varying environmental conditions which may cause the structures to suffer from fatigue damage and/or failures, and thus require real time structural health monitoring (SHM). The diagnostics process and condition monitoring of composite structures is usually carried out during their working life [2]. The goal of such diagnostics is to detect, identify, locate and assess the defects that may affect the safety or performance of a structure. Sensors that are commonly employed for SHM are resistance strain gauges, fiber optic sensors, piezoelectric sensors, eddy current sensors, and micro-electromechanical systems (MEMS) sensors [3]. Traditional nondestructive evaluation techniques such as ultrasonic inspection, acoustography, low frequency methods, radiographic inspection, shearography, acousto-ultrasonic and thermography are effective for SHM of composite materials and structures, but frequently it is difficult or impossible to use such nondestructive evaluation techniques in an operational structure due to the size and weight of the systems [4]. Fiber optic sensors (FOS) on the other hand are suitable candidates for SHM of composite materials during operation since they are capable of achieving the goals of diagnostics as well as condition monitoring and being very compact in size and can also be embedded into such structures, acting in many ways as the equivalent of a human nervous system [5]. Previous investigations of FOS embedded in composite structures indicate that FOS technology is capable of monitoring stress/strain, temperature, composite cure process, vibration, humidity, delamination and cracking and thus has great potential for condition monitoring of a variety of composite materials applications [6,7].

Recognizing the increase in the use of composite structures and the need for them to perform “smart” functions, such as sensing and actuation, this paper for the first time presents a comprehensive overview of recent advances in the area of FOS technology for condition monitoring involving strain and temperature measurements in composite materials and also quantities such as thermal expansion. An overview of different types of most commonly used composite materials and their properties is given in Section 2. Then the demand for the SHM in composite material structures is discussed in Section 3, following which the common fabrication methods of composite materials with embedded fiber sensors are detailed in Section 4 while the issues of composite degradation associated with embedding of fiber sensors are discussed in Section 5. An overview of different types of fiber sensors for strain/temperature measurements in composite materials is presented in Section 6. The recent trends, issues and future challenges of the FOS technology are discussed in Section 7.

## 2. Composite Materials and Demand for the SHM in Composite Material Structures

In this section we discuss the various classifications of typical composite materials and their properties. In general, fibre composite materials have two constituent materials; reinforcement fibres and a matrix [8] that when combined together can produce a material with properties superior to those of the constituent materials. It is known that the mechanical properties of a composite material differ depending on the matrix and the reinforcing materials used to fabricate the composite [8,9]. The functions of the matrix in a composite material are: to transfer the load to the reinforcement fibres; to provide temperature resistance and chemical resistance and to maintain the reinforcement fibres in a fixed orientation [10].

The second constituent of a composite material is the reinforcement fibre [9]. The composite material’s tensile properties, stiffness and impact resistance are influenced by the type of the fibre reinforcement [10]. As regards applications, fibre reinforced composite materials are commonly used for the fabrication of various structural parts such as aircraft tails, wings, fuselages, propellers, helicopter rotor blades, wind turbine blades, in the construction industry and as components in racing cars, boats, *etc.* [7]. One of the key challenges in the design of a composite structural part in many applications is the need to achieve a fail-safe design solution, which requires optimisation of multiple parameters. Typically given the performance specifications for a composite part [9], the areas that need optimisation are: the selection of most appropriate reinforcement and matrix constituents to satisfy the requirements for strength and stiffness of the particular composite part with a minimum weight; the selection of the composite part’s geometry; a careful analysis of stress distribution within the part; the minimization of moisture ingress; optimization of toughness and an analysis of failure modes. Prior to opting for a particular design for mass production the manufacturer also needs to consider the repeatability of the composite part’s performance specification, ease of production, cost efficiency and quality assurance mechanisms [10,11,12]. Finally, even though the designed structural part is optimised for fail-safe performance there is a possibility of damage during operation in extreme environmental conditions and mechanical failure of the structure due to external perturbations [3] and this necessitates the requirement for non-destructive structural health monitoring techniques throughout the lifetime of the composite structural part.

**Figure 1:**
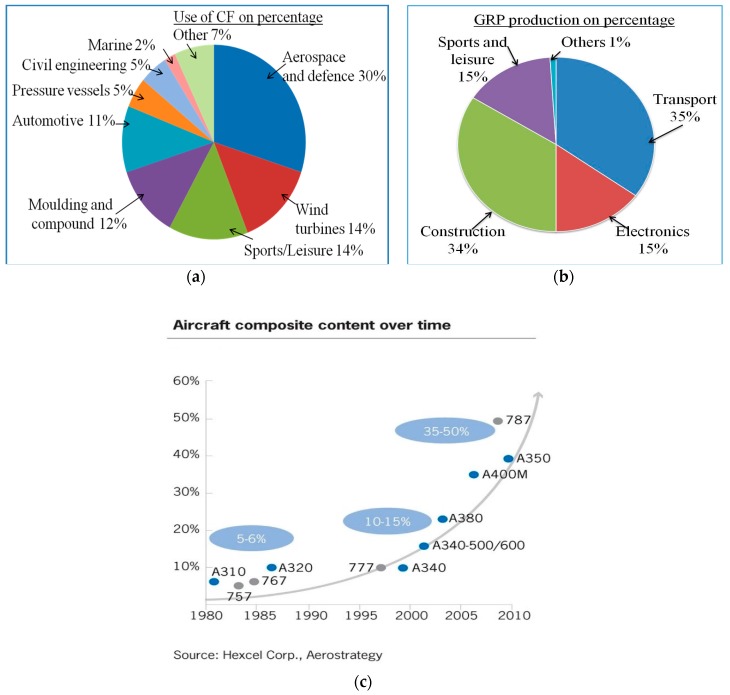
(**a**) Use of CF composites by industry [14]; (**b**) GRP production in Europe for different application industries [14] and (**c**) development of composite aerospace applications in last 40 years (source data from *Hexcel Corp. Aerostrategy*).

The demand for SHM in composites is in the first instance driven by the increased use of composite materials. The widespread use of carbon reinforced fibre plastics (CRP) and glass fibre reinforced plastic (GRP) composite based structural parts has significantly increased in application areas such as aircraft, sport vehicles, wind turbines, and infrastructure constructions, because of the inherent advantages of composite materials. For example, in aircraft the advantages are well known: lighter weight for the aircraft, reduced requirements for maintenance and increased passenger comfort [13]. The use of composite materials in aircraft is growing, as shown in Figure 1a–c. Aircraft that utilize a high proportion of lightweight composite materials consume less fuel and result in a high cost benefit that yields energy savings up to 18%. Some existing commercial aircraft models such as the Boeing 787 and Airbus A350 are comprised of *circa* 50% composite materials by weight. Market research conducted in 2013 by Jahn and Witten indicates that there will be a growing demand for such aircraft in the coming years [14]. Figure 1a,b show the overall use of CFP composites by different industries and GRP production in different European industries respectively.

The driving force behind the need for SHM in composite materials is the necessity to monitor composite parts during operation, for safety and early detection of failure. During a typical 20-year service life, composite structures such as wind turbine blades, helicopter blades, construction parts and aircraft parts are subjected to static and dynamic lift, drag and inertial loads over a wide range of temperatures and often severe environmental conditions. As the growth in production and use of composite parts has increased, the composite industry has increasingly focused on damage/failure free composite structures and non-destructive techniques for SHM over a part’s life time. Compared to conventional non-destructive sensing techniques, optical fiber sensors have achieved wide acceptance due to their attractive properties such as small size, immunity to electromagnetic interference and low cost [1]. Optical fiber sensors embedded in various structures are very useful for strain/temperature monitoring [2] applications in extreme environmental conditions. For example, issues such as bend loading in aircraft wings and bridges can be monitored and avoided by implementing smart composite structures with embedded fiber optic sensors. Also more complex sensing needs can be met, such as the detection of the formation of ice on the wings of an aircraft by implementing smart composite structures with embedded fiber optic sensors within the wings material. Monitoring of the wing bend or loading due to ice accumulation combined with temperature data can improve the efficiency of the aircraft’s de-icing systems. As a result, it can be concluded that smart composite materials with embedded fiber optic sensors can significantly enhance the safety of advanced machines, structures and devices.

## 3. Composite Materials with Embedded Fiber Sensors: Fabrication Methods

In this section we discuss the commonly employed sample fabrication methods for composite materials with embedded FOS.

### 3.1. Fabrication Method of Composite Samples Embedded with FOS by Hand Layup and Pre-Preg Layup Methods

The most common fabrication processes adopted by laboratory based and small scale industrial manufacturers are the expertise intensive hand layup and pre-preg layup [15] methods. The hand layup method is a process for arranging fiber–reinforced layers in a laminate and shaping the laminate to fabricate the desired part. For this the reinforcement fibers or fabrics are stacked one over another by applying the matrix in between them. After stacking, curing (or polymerization) of the resin matrix of the multiple laminate layered composite, the sample can be shaped as per the manufacturer’s specifications. Pre-pregs are single laminates of “pre-impregnated” composite fibers with a matrix material such as epoxy resin. In the pre-preg layup method, multiples of composite pre-preg laminates are stacked one over another and the curing can be done unaided or by applying heat and/or pressure. The moulding process can be either vacuum—bag moulding, or an autoclave moulding. Typical autoclave curing conditions involve a temperature range from 120 °C to 200 °C with an applied pressure up to 100 psi (~6.89 kPa).

**Figure 2:**
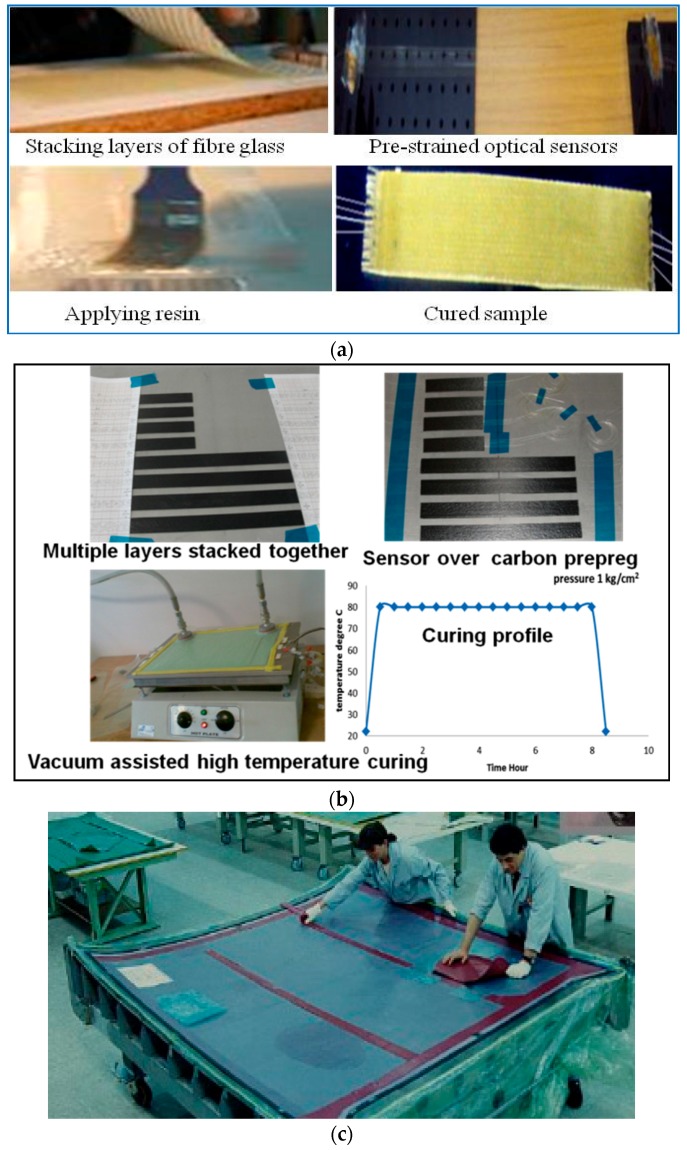
Embedding fiber sensors inside composite materials (**a**) hand layup; (**b**) pre-preg method, and (**c**) expert assisted manufacturing of composite part embedded with FOS.

For embedding optical fibers in samples prepared by both the hand—layup and the pre-preg layup methods the process is similar as follows: before the curing process optical fibers are placed on the corresponding composite layer and some pre-strain is applied to make sure the optical fibers remain free of bends [16]. Positioning of the optical sensor is highly application specific and depends on the location of the areas where parameters need to be monitored. For example, for applied strain and temperature measurements of composite material structures it is reported that optical fiber sensors were embedded within the furthest layer from middle layer to achieve the highest measurement sensitivity [17]. The different steps for embedding optical fiber sensors by the hand layup and pre-preg methods are shown in Figure 2a,b respectively. For larger composite parts, optical fibers can be embedded manually with expert assistance as shown in Figure 2c, but for large scale production of smart composite structures materials embedded with optical fiber sensors automated robotic systems can be used. One of the most important concerns while embedding FOS inside composite structures is their influence on the structural integrity of the composite part. In the next section we have detailed some methods to reduce the risk of composite material degradation and to maintain the structural integrity of the composite material structures with embedded sensors.

### 3.2. Rotating Filament Wound Pressure Cylinder with FOS

Fabrication details of E-glass fibre reinforced composite based pressure cylinders with FOS was reported earlier [18] for the composite parts with cylindrical shapes. In this method a machine assisted filament winding process in the circumferential direction is used. Continuous E-glass roving reinforcement fibre with different orientation angles can be used to achieve a defined length for the fabricated pressure cylinder. The winding angle ±55° with respect to the cylinder’s long axis is the classical winding angle for pressure vessels, where circumferential stress is twice the value of axial stress. The machine assisted filament winding process operates in such a way that at both ends of the cylinder there are zones of reinforcing fibers, placed at an angle of 90° to the cylinder axis. For embedding FOS in to the composite cylinders, a specifically designed mandrel with a winding machine rotary is co-operated with a rotating filament winding machine. The matrix material is applied during the winding process. The matrix material used is a mixture of Araldite LY 5052 epoxy and hardener HY 5052. The samples are cured at temperature 50 °C for 900 min. More detailed explanation of this particular method is given in reference [18].

Fabrication method for a pressure cylinder made of carbon fibre reinforced epoxy pre-preg embedded with FOS was reported in [19]. In this method the carbon fibre reinforcement is wound using machine assisted rotating filament. The optical fibre is embedded between the first and second plies of the laminate sequence. Pre-stressed optical fibres are drenched in epoxy resin and are wound onto a water-soluble mandrel after which the vessel is cured in an oven. The winding of the pressure vessels consists of five layers in total: in which two polar windings with winding angles of 12.7° and 16.5° and three hoop windings with a winding angle of approximately 90°. The optical fibre is carefully embedded during the winding process of the vessel in the outer 90° winding, parallel to the reinforcing fibres.

### 3.3. Composite Panels Embedded with FOS

A laboratory level fabrication method of composite panels with embedded FOS using specially designed Resin Transfer Molding (RTM) apparatus is reported earlier [20,21,22]. The specially designed RTM apparatus allows for different types of mold designs with variable mold thicknesses. Automatic control system is used for the resin injection process and a transparent glass top is provided for visual monitoring of resin flow. Automated control of curing temperature as well as the mold part temperature is achieved by using thermocouple sensors with an automated water temperature controller. The FOSs are embedded into the perform itself. The ingress/egress parts of optical fibers that enter in to the composite panels are at an angle close to 90° relative to the component’s surface. A thin bended hypodermic tube is placed around the optical fiber such a way that the bend radius of the hypodermic tube is carefully chosen to avoid the possibility of fiber fracture. To protect the optical fibers from the disturbances due to reinforcement preform, a tapered silicon stopper is fitted in a hole in the mold and the hypodermic tube passes through this stopper. Thus the ingress/egress is sealed and is capable of withstanding resin injection pressure of approximately 0.75 bar.

### 3.4. Braided Composites Embedded with FOS

A method to co-braid optical fibers into braided composites is also reported [23]. The reported advantage of this method is that the embedded optical fibers will not cause large resin pockets in the material. In this method, optical fibers are incorporated as an axial yarn so that the optical fibers run straight as well as parallel with respect to the specimen braiding direction. The structural reinforcement fibres are then braided around the optical fibers. The types of FOS that are reported incorporated with such braided composites are the Fabry-Perot fiber optic sensor, polarimetric optical fiber sensor, and the Bragg grating fiber optic sensor. A special RTM molding dye is designed to protect the optical fibers from any damage during the curing process. The RTM process is used for composite fabrication using a release agent, Epoxy 828 resin, and a curative. The temperature of the molding process is 85 °C, at the pressure of 0.1 MPa. Carbon fibre is used as reinforcement and the specimens are braided with a fiber volume fraction of 50%.

### 3.5. FOS Stitched Carbon Fibre Preforms for Advanced Composite Structures

Incorporating optical fibers during carbon fibre based preform fabrication itself is promising and has been reported earlier [24]. In this method optical fibers were stitched into each fiber preform along the middle plane of the laminate. FOS stitched carbon fibre preforms were successfully demonstrated for condition monitoring as well the resin advancement monitoring through the preform during the infusion. For monitoring the resin advancement, a reference optical fiber was placed as near as possible to the infusion tube in order to estimate the entry time of the resin as a reference time for the FOS signals [24].

## 4. Composite Material Degradation Associated with Embedding of Fiber Sensors

One of the major concerns when embedding FOSs in composite materials is the degradation of the composite material’s mechanical properties and also the possible increase in the probability of failure due to the presence of embedded optical fibers [25]. Various studies have been carried out to analyse the influence of the embedded FOS on composite material tensile/compressive strength, stiffness, inter laminar fracture toughness, and fatigue resistance, *etc.* It is reported that having embedded optical fibers passing through or parallel to ply-drops in a laminate does not have any significant effects on the static strength of the laminate [26,27]. This is valid even for optical fibers placed in the most critical locations. In principle any potential degradation of strength and the modulus of a composite material will be a function of the orientation of the optical fiber relative to the nearest plies, the overall thickness of the laminate, the optical fiber diameter, and type of protective coating on the optical fiber [26]. Degradation becomes increasingly severe with an increasing angle between the optical fiber and ply directions. Another issue is the larger diameter of the optical fiber (with its buffer coating) compared to the ply fibers of the composite. In general, commercially available optical fibers have diameters from 125 µm to 230 µm, which is about ten to fifteen times larger than the average E-glass fiber or carbon fiber. Embedding the optical fiber perpendicularly to the direction of the reinforcement fibres can result in appearance of characteristic “eye” patterns or “pockets” within the resin, which act as defect centers in the composite part that could ultimately lead to premature failure in the form of delamination [27]. However, testing conducted at a number of labs worldwide concluded that such delaminations were insignificant if the FOS density was low. It reported that if the optical fiber is laid along the direction of reinforcement fiber there is a uniform consolidation around the optical fiber with minimum defects and thus the laminate’s mechanical parameters are least affected [6,26,27,28,29]. Also the optical fibers laid along the direction of fiber reinforcement have limited influence on the mechanical behavior of composite structure because of optical fiber’s own inherent load carrying ability [26].

An analysis [6] on the flexural strength of a composite material, embedded with FOSs, that bears tensile loading showed that the flexural strength did not suffer any noticeable degradation when the FOS were embedded in the tensile region either in the longitudinal direction or in the transverse direction. For compressive loading, the situation is similar if the sensor is embedded in the longitudinal direction but it is found that if the FOS is embedded in the transverse direction with respect to the compressive region, the flexural strength is degraded significantly [30]. In order to realize SHM for composite material components in many applications it is essential to measure the strain and temperature of composite materials and in the next section we discuss the different types of FOS strain and temperature sensors that can be embedded in composite materials.

## 5. Types of Fiber Optic Sensors for Strain/Temperature Measurements in Composite Materials

There are a wide variety of applications of fiber optic sensors in composite materials, including vibration measurements, cure process monitoring, temperature measurements, thermal expansion measurements, detection of delamination/debonding, three dimensional strain measurements, thermal strain measurements, relative humidity measurements and detection of cracking, *etc.* [31,32,33,34]. For all these applications the key requirement is to measure either strain or temperature or both parameters. In this section we discuss the different type of FOS that can be used with composite materials to measure strain/temperature when embedded inside a composite material. The different types of FOS reported for strain/temperature measurements in composite materials are fiber Bragg grating (FBG) sensors [35,36,37], interferometric fiber optic sensors [38], polarimetric sensors [39], fiber optic micro bend sensors [40], distributed sensors (using techniques such as Rayleigh scattering, Raman scattering, and Brillouin scattering) [41,42] and hybrid sensors [43,44,45]. The aim of this review is to provide essential information for the smart materials industry in selecting of appropriate types of FOS in accordance with the end-user requirements and applications.

### 5.1. Fiber Bragg Grating Sensor for Composite Materials

**Figure 3:**
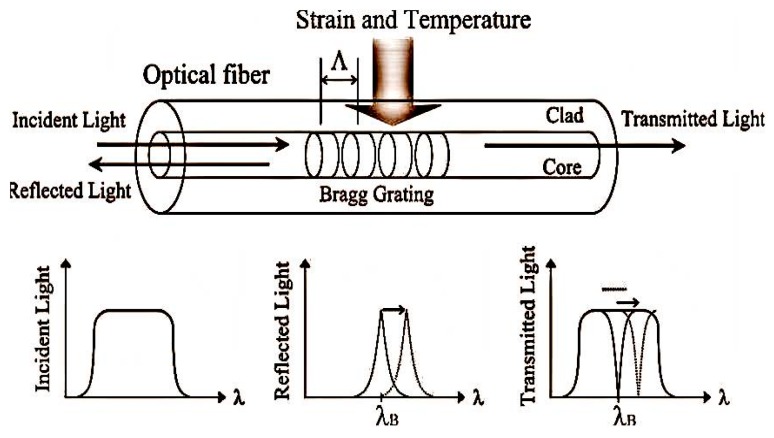
Fiber Bragg grating.

FBGs are the most commonly employed fiber optic sensors in SHM applications for composite materials [35,36,37]. A fiber Bragg grating sensor comprises of a grating region with a periodic change in refractive index in the core region of an optical fiber. Such a periodically modulated refractive index structure enables the light to be coupled from the forward propagating core mode into a backward propagating core mode generating a reflection response, as shown in the schematic illustrating the principle of an FBG provided in Figure 3 [46]. The light reflected due to periodic variations of the refractive index of the Bragg grating with a central wavelength given by [46]:

(1) $$\lambda_{B}=2n_{0}\Lambda$$

where *n*_0_ is the effective refractive index of the guided mode in the fiber core and Λ is the grating pitch.

The strain sensitivity of the Bragg wavelength arises from the change in the pitch of the fiber grating due to strain and changes in the refractive index arising from the strain-optic effect. When a strain is applied to the grating, the Bragg reflected wavelength changes. The wavelength shift Δλ_ε_ for a value of elongation Δ*L* is given by [46]:

(2) $$\Delta \lambda_{\varepsilon }=\lambda_{B}\cdot \left(\frac{1}{\Lambda }\frac{\partial \Lambda }{\partial L}+\frac{1}{n_{0}}\frac{\partial n_{0}}{\partial L}\right)\cdot \Delta L$$

In practice the applied strain value can be estimated from the measurement of reflected wavelength as it changes due to applied strain. The typical strain sensitivity of an FBG at 1550 nm is ~1.2 pm/µε.

Bragg wavelength shift can also occur due to changes in temperature. For a temperature variation of Δ*T*, the corresponding wavelength shift Δλ*_T_* is given by [46]:

(3) $$\Delta \lambda_{T}=\lambda_{B}\cdot \left(\frac{1}{\Lambda }\frac{\partial \Lambda }{\partial T}+\frac{1}{n_{0}}\frac{\partial n_{0}}{\partial T}\right)\cdot \Delta T$$

**Figure 4:**
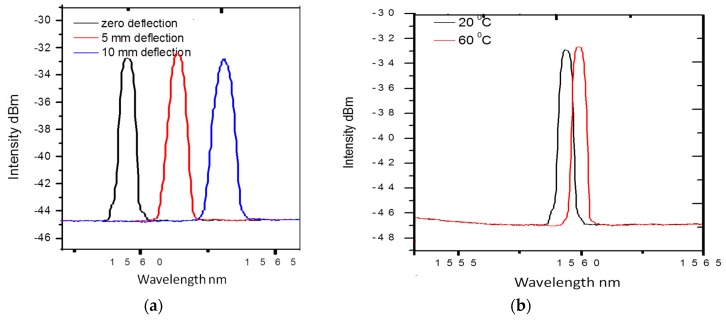
(**a**) Measured wavelength shift for the FBG sensors at different deflection values (**b**) measured wavelength shift for the FBG sensors at different temperatures [56].

The temperature sensitivity of the Bragg wavelength arises from the change in the grating pitch associated with the thermal expansion of the fiber, and the change in the refractive index arising from the thermo-optic effect. Thus Equation (3) can be also written as [46]:

(4) $$\Delta \lambda_{T}=\left(\alpha_{0}+\beta_{0}\right)\cdot \lambda_{B}\cdot \Delta T$$

where α_0_ is the coefficient of the thermal expansion (CTE) of the fiber, and β_0_ is the fiber refractive index variation with temperature, respectively. The values of α_0_ and β_0_ are constants for silica optical fiber, and are 0.55 × 10^−6^/°C, and 6.6 × 10^−6^/°C, respectively. The typical temperature sensitivity of an FBG at 1550 nm is ~11.6 pm/°C. Commonly FBGs are used to measure axial strain as well as temperature. This is because axial strain sensitivity is effectively higher as the change in the FBG pitch is directly proportional to the applied longitudinal strain. The measured wavelength shift of the embedded FBG sensors for different deflection values during a three-point bending strain test on composite material is shown in Figure 4a. The measured wavelength shift of the embedded FBG sensors at different temperatures of composite materials is shown in Figure 4b. One can see that a red shift of the Bragg wavelength arises as a result of an increase in temperature. Other than standard Bragg gratings with a uniform period, there are chirped gratings with a gradual period variation [47], and tilted fiber Bragg gratings (TFBG) [47] with gratings written at an angle to the fiber axis are also employed for strain temperature measurements in composite materials. The chirped FBG sensor has a gradual distribution of the grating period [48]. This variation in the grating period provides a one-to-one correspondence between the wavelength in the spectrum and the position of the gauge section within composite material, which is the significant advantage of chirped FBG sensors over the conventional FBG sensors [48]. The main disadvantage of conventional FBG sensors is the cross-sensitivity between temperature and strain. In a different manner from conventional FBGs, the wave vector of a TFBG has a certain angle with respect to the fiber axis; making the resonance wavelengths of core mode and cladding mode to be sensitive to temperature, while their transmission power is temperature-independent [49]. Thus based on these unique characteristics of TFBG, it is possible to achieve simultaneous discrimination of mechanical perturbations and temperature [47,49]. Different methods have been reported by other authors to compensate for the cross-sensitivity effects between temperature and strain; these methods include introduction of a reference FBG [50], dual-wavelength superimposed FBGs [51], different cladding diameter FBGs [52], combined FBG and a long-period grating [53], combination of an FBG and a Fabry-Perot interferometer [54], and superstructure FBG method [55].

It has also been reported that for coating stripped FBGs thermal expansion of a composite material as well as the non uniform strain can cause Bragg peak distortion and broadening [28,56]. The strain components acting in different directions within the composite structure can be measured using multiplexed FBGs [57]. In addition, a non-isotropic strain distribution within the composite material can be measured by using an FBG written in a micro-structured high birefringence fiber [58].

#### 5.1.1. FBG Written in Micro-Structured Fibers as a Sensor for Composite Materials

**Figure 5:**
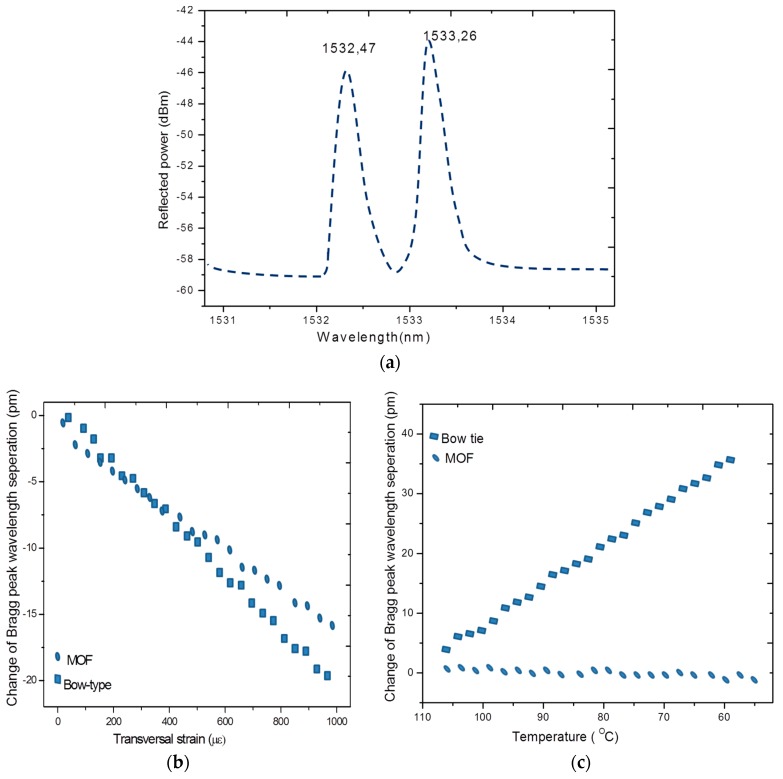
(**a**) Reflection spectra for an FBG written in a HB-PM-PCF with two peaks corresponding to slow axis and fast axis; (**b**) change in the peak separation with transverse strain for FBGs written in MOF and bow-tie type fibers; and (**c**) change in peak separation with temperature [60].

A conventional FBG sensor’s primary response is to an axial strain but the transverse strain also has some influence on the FBG sensor’s response [57,59]. But it is difficult to discriminate axial strain and transverse strain from FBG spectral response. For strain mapping in some SHM applications, for example, for the detection of damage, cracks, delamination etc a multi axial strain measurement is required. This in turn results in the importance of development of a sensing scheme that provides measurements of axial strain together with transverse strain. It is reported that FBGs written in the highly birefringent (HB) fibers and high birefringent micro-structured fibers (HB-MOF) have real potential to measure transverse strain and axial strain simultaneously [58,60]. As represented in Figure 5a, an FBG written in a highly birefringent fiber displays two Bragg peaks, corresponding to both orthogonally polarized modes. The change of the Bragg peak separation depends on the phase modal birefringence variation induced by transverse load and temperature. The properties of an MOF and its sensitivity to different measurands are determined by the type of the fiber used. However it is reported that FBGs written in HB fibers such as the bow-tie type have the disadvantage of greater temperature and strain cross sensitivity [59].

The cross-sensitivity issue can be resolved by writing FBGs in low temperature sensitive MOFs, such as high birefringent polarization maintaining photonic crystal fibers (HB-PM-PCF). For example, a comparison response of the embedded FBG sensors written in conventional birefringent optical fibers (bow-tie) and a HB-PM-PCF when the composite material is exposed to controlled mechanical and thermal loads are presented in Figure 5b,c, respectively. For the FBG in a bow-tie type fiber the Bragg peak separation varies in accordance with the transverse strain and temperature as shown in Figure 5b,c. However in the case of an FBG in a low temperature sensitive MOF, the Bragg peak separation varies with transverse strain only. A temperature independent axial strain and transverse strain measurement in a composite material can be carried out using an FBG written in a HB-PM-PCF [60]. It is also reported that the two Bragg peaks in the case of an FBG written in HB-PM-PCF are farther separated than for an FBG in a bow-tie fiber and therefore FBGs written in MOF/HB-PM-PCF allow for more accurate measurements of the peak wavelengths and thus are more suitable for composite material sensing applications despite high splice loss induced lower reflectivity of the grating [58,60].

#### 5.1.2. Phase-Shifted FBGs for Composite Materials

**Figure 6:**
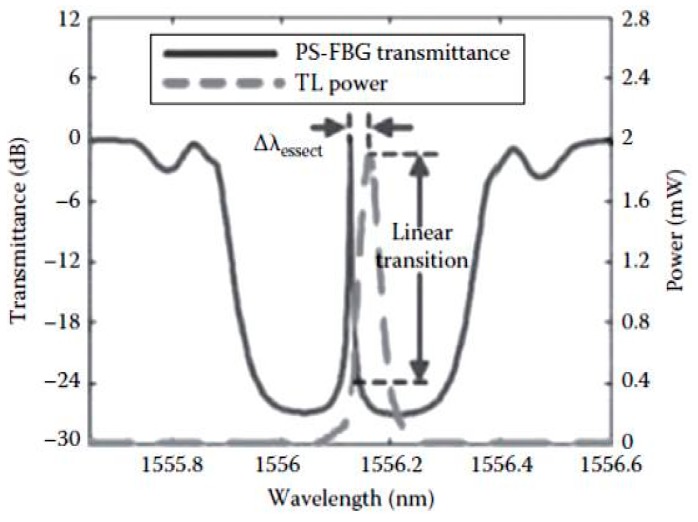
Spectral response of a PS-FBG and its interrogation technique based on a narrow band laser signal [61].

The phase-shifted FBG (PS-FBG) sensor together with its interrogation system is proven as one of the high sensitivity methods for acoustic emission (AE) measurement [61]. PS-FBGs are widely used in optical fiber communications and optical fiber sensing applications as a wavelength multiplexer and also as a strain sensor. The extremely sharp resonance in the transmission spectrum of the PS-FBG enables it to develop a high sensitivity interrogation system capable of measuring very small strain changes, even at high acoustic frequencies. A typical transmission spectrum of a PS-FBG interrogated using a tunable laser is shown in Figure 6. It has been demonstrated that using this method microscale strains can be measured and thus the sensor is suitable for AE sensing in composite structures [61].

#### 5.1.3. Polymer FBG Sensor for Composite Materials

For composite materials, the attractive characteristics of polymer FBGs include their high temperature sensitivity, large strain range, and the absence of buffer coating [62,63,64]. These distinct features of the polymer FBGs might provide some advantages over standard silica FBGs in measuring some of the parameters of the composite materials.

**Figure 7:**
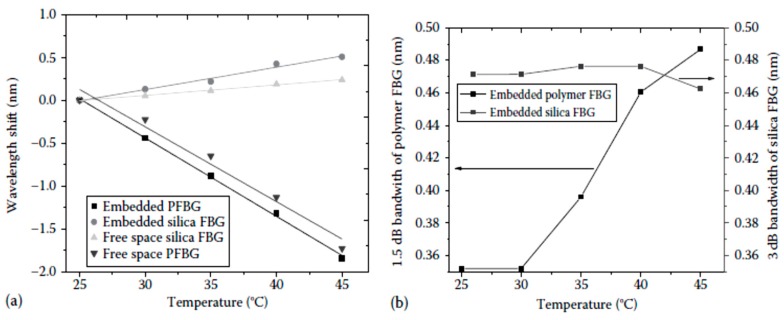
(**a**) Temperature-induced wavelength shift of the embedded polymer and silica FBGs and its comparison with free-space FBGs; (**b**) measured 1.5 dB bandwidth of polymer FBG and 3 dB bandwidth of silica FBG at different temperatures [65].

**Figure 8:**
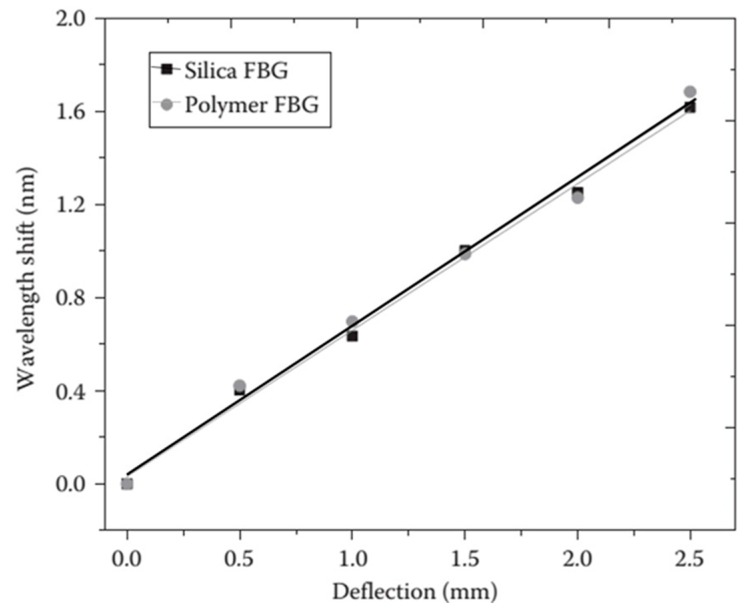
Wavelength shifts of the polymer and silica FBGs with deflection in the middle of the composite material [65].

Rajan *et al.* conducted studies with polymer FBGs embedded in glass-reinforced composite materials [65]. For the polymer FBG embedded in composite materials, due to temperature change, spectral broadening is observed together with the shift in the peak reflected wavelength. The observed wavelength shifts of the polymer FBG embedded in a glass-reinforced composite and its comparison with silica FBG and free-space FBG sensors are shown in Figure 7a. A blue shift in the wavelength is observed for the polymer FBG due to the negative thermo-optic coefficient, while a red shift is observed for the silica FBG. The observed temperature sensitivity of the embedded polymer FBG is −92.28 ± 2 pm/°C, which is close to the free-space temperature sensitivity of −90 ± 6 pm/°C. The change in the bandwidth of the reflection spectra of the polymer FBG at different temperatures and its comparison with silica FBG is shown in Figure 7b. It can be seen that the bandwidth of the polymer FBG increases as the temperature increases. The measured bandwidth change for the embedded polymer FBG within the temperature range of 30 °C–45 °C was 8.5 pm/°C. Therefore, from the observed spectral broadening and distortion, it can be concluded that the thermal expansion induced stress is effectively transferred to the polymer fiber and can be measured using a polymer FBG. It is assumed that the reason for this is the absence of a buffer coating for the polymer fiber, which results in a direct transfer of the surrounding physical phenomena to the core and cladding of the polymer fiber. Stress induced by localized microbends in the composite material could also contribute to the chirping effect. The strain sensitivity of the embedded polymer FBG and its comparison with silica FBG is shown in Figure 8. From the figure, it can be seen that the strain sensitivity of the embedded polymer and silica FBGs are very close in value to each other. In free space, the polymer FBG had a slightly higher strain sensitivity (1.340 ± 0.015 pm/με) compared to that of silica FBG (1.2 ± 0.01 pm/με). The similarity in the measured strain sensitivity of the embedded silica and polymer FBGs underlines the fact that longitudinal strain in the composite is not effectively transferred to the polymer fiber. This can be attributed to the differences in mechanical properties of the polymer fiber and the composite material, which resulted in the mechanical strain not being effectively transferred to the polymer FBGs as compared to the case of silica FBG. With further works in this area, polymer FBG has the potential in measuring temperature and thermal expansion of the composite material simultaneously.

### 5.2. Interferometric Fiber Sensors for Composite Materials

Interferometric fiber sensors [66] can also be employed for strain/temperature measurements in composite materials and this is discussed in detail in this section. There are different types of interferometric fiber sensors which differ in their operating principles and strain/temperature sensing characteristics [38]. Among the interferometric fiber sensors most commonly employed for composite sensing applications are: extrinsic Fabry-Perot interferometers (EFPI), micro-hole collapsed modal interferometers and Sagnac fiber loop mirror (FLM) sensors.

**Figure 9:**
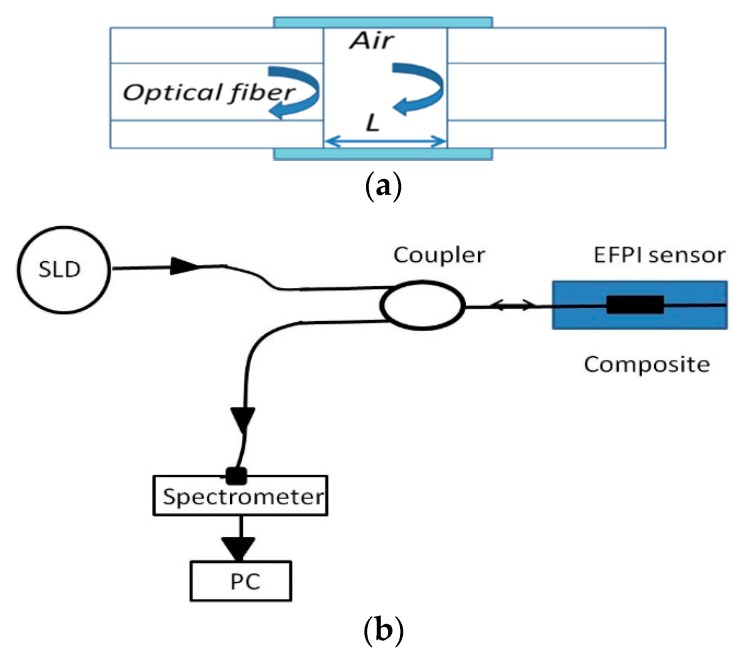
(**a**) One of the typical EFPI sensor design; and (**b**) schematic experimental arrangement for the EPFI sensor [70].

A Fabry-Perot interferometer (FPI) generally comprises of two parallel reflecting surfaces separated by a certain distance [66]. One of the EFPI sensors is illustrated in Figure 9a. In an extrinsic FPI sensor, the interference occurs due to the multiple superpositions of both reflected and transmitted beams at two parallel surfaces [38,66]. It is possible to tune the intensity of the interferometric modes of an EFPI sensor by varying the gap between the two reflecting surfaces [66]. The reflection or transmission spectrum of an FPI is a wavelength dependent intensity modulation of the input light spectrum, resulting from the optical phase difference between the reflected and transmitted beams [66,67]. The maxima and minima of the modulated spectrum indicate that both reflected and transmitted beams, at that particular wavelength, are in 2π phase and 2π out-of-phase, respectively. The optical phase difference between reflected or transmitted beams at a particular wavelength of the FPI is basically specified as [66]:

(5) $$\delta =\left(\frac{2\pi }{\lambda }\right)n2L$$

where λ is the wavelength of incident light, *n* is the refractive index (RI) of cavity material, and *L* is the length of the cavity.

**Figure 10:**
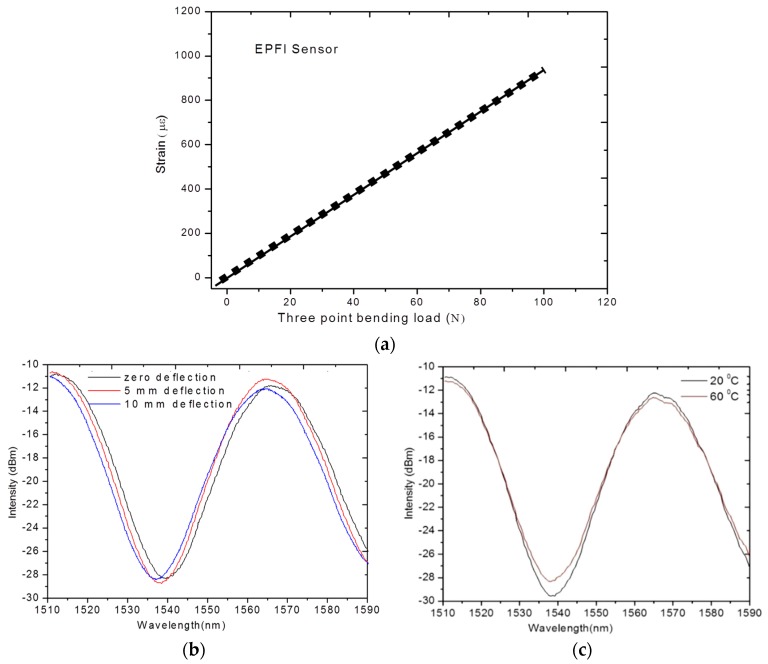
(**a**) Experimentally measured strain using embedded EFPI sensor during three point bending test in a composite [69]; (**b**) Responses of the of photonic crystal fiber based sensors embedded in the composite material sample during deflections based on three point bending test [70]; and (**c**) at different temperatures of the composite sample [56].

The schematic experimental arrangement of the EPFI sensor is as shown in Figure 9b, which comprises of a superluminescent light diode (SLD) source, a coupler and a spectrometer. Any applied longitudinal strain to the FPI sensor alters the physical length of the cavity, which results in a phase difference between reflected or transmitted beams. By measuring the shift of the wavelength spectrum, the strain applied to the FPI can be measured. It is found that the shorter the optical path difference (OPD) the larger will be free spectral range (FSR), resulting a wider dynamic range for a sensor [38,67]. Therefore, the dynamic range of the sensor can be tuned by varying the cavity length which in turn changes the OPD of the FPI sensor [68]. Figure 10a illustrates experimentally measured strain using embedded EFPI sensor during the three point bending test of a composite material sample [56]. Moreover, EFPI sensors based on photonic crystal fibers have proved to be good candidates for measurements of axial strain in composite materials since their sensitivity to temperature is insignificant [38,69].

Recent advancements in the area of photonic crystal fiber (HB-PM-PCF) sensors [38,56] have opened new possibilities for the development of temperature insensitive micro-hole collapsed photonic crystal fiber modal interferometric sensors. Micro-hole collapsed photonic crystal fiber modal interferometers can be used for localised strain measurements in a composite material. Micro-hole collapsed photonic crystal fiber modal interferometers can be fabricated by fusion slicing of a HB-PM-PCF section between two standard single mode fibers. During the splicing process, at both ends of the HB-PM-PCF section, the holes of the HB-PM-PCF are collapsed in a microscopic region. The first collapsed region allows for the excitation of multiple modes in the HB-PM-PCF and at the second collapsed region at the other end the modes recombine and thus the HB-PM-PCF section forms an interferometer [56]. It is reported that temperature independent strain measurement in composites is possible using such a micro-hole collapsed photonic crystal fiber interferometer fabricated from a micro–structured HB-PM-PCF fiber (LMA-10) [56]. An example of the output of a micro-hole collapsed modal interferometer embedded in composite beam undergoing three point bending deflection is shown in Figure 10b [69]. Figure 10c illustrates that such a sensor embedded in composite material has very low temperature dependence [56].

**Figure 11:**
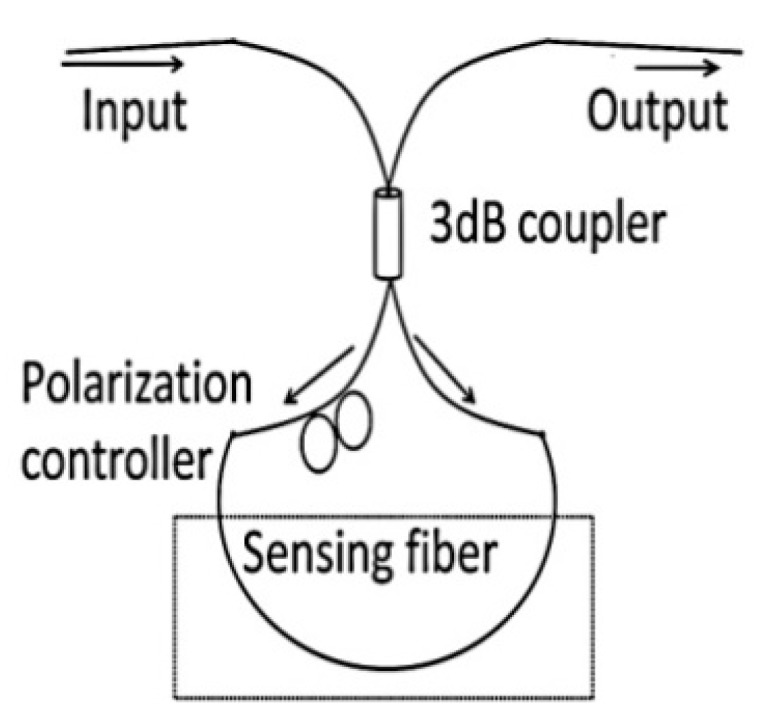
Schematic of the sensor based on a Sagnac interferometer.

Fiber optic Sagnac interferometers (SIs) are another promising candidate for composite material sensing applications. In a fiber optic SI input light is split into two parts propagating in the opposite directions by a 3 dB fiber coupler and these two counter-propagating beams are combined again at the same coupler as shown in Figure 11 [66]. The fabrication of such an interferometer can be simply achieved by connecting the ends of a conventional 3 dB coupler. High birefringent fibers (HB) or polarization maintaining fibers (PMFs) are typically utilized as sensing fibers since HB fibers maximize the polarization-dependence of the signal within the SIs. In order to control the input light polarization a polarization controller (PC) is connected to the sensing fiber. The signal at the output port of the fiber coupler is a result of interference between the beams polarized along the slow axis and the fast axis. The phase of the interference is simply given as:

(6) $$\delta_{SL}=\frac{2\pi }{\lambda }BL$$

where *B* = |*n_f_* − *n_s_*| is the birefrigent coefficient of the sensing fiber, *L* is the length of the fiber, *n_f_* and *n_s_* are the effective indices of the fast and slow modes.

**Figure 12:**
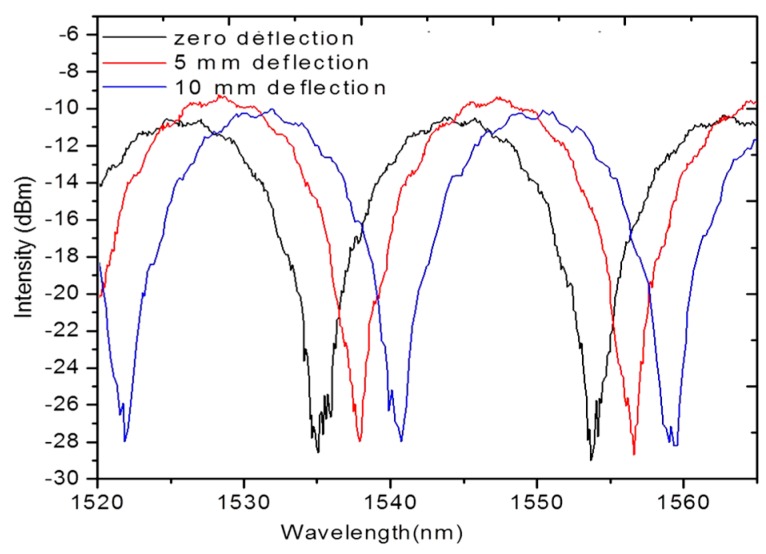
Responses of the HB-PM-PCF based SI sensor embedded in the composite material sample during deflections based on three point bending test [56].

Fiber optic-based SIs can be used for measuring parameters such as strain, temperature, pressure, twist, *etc.* Such fiber optic based SIs provide the value of the sensed parameter averaged over the length of the sensor. However, a disadvantage of the SI is its significant temperature strain cross-sensitivity [66]. It is reported that the temperature strain cross-sensitivity issue can be eliminated by employing low temperature sensitive HB-PM-PCF [56]. The output of a low temperature sensitive HB-PM-PCF based SI sensor embedded in composite beam undergoing three point bending deflection is shown in Figure 12 [56]. Such low temperature sensitive high birefringent fiber based SIs are an appropriate option for measuring strain acting on a composite material over the length of the sensing fiber.

### 5.3. Optical Fiber Polarimetric Sensors for Composite Materials

The polarization properties of light propagating though an optical can be affected by stress, strain, pressure and temperature acting on a measuring fiber and in a fiber polarimetric sensor, the polarization change is detected to retrieve the sensing parameter [71,72]. A symmetric deformation effect or temperature variation in a single-mode fiber influences the propagation constant (β) for every mode because of the changes in the fiber length (*L*) and the refractive indices of the core and the cladding [39,73]. Under the influence of a longitudinal strain (ε) and at a constant temperature, for polarimetric sensors the change in the phase difference can be written as [73]:

(7) $$\delta (\Delta \Phi )=\frac{\partial (\Delta \beta )}{\partial \varepsilon /T}\delta L$$

Fiber optic polarimetric sensors can be realized by different types of PM fibers such as Panda fiber, bow-tie type fiber, side-hole fibers and PM-HB MOF fibers. Fiber optic polarimetric sensors can be embedded in composite materials to measure the average strain/temperature over the sensor length [39,73]. It is possible to vary the strain/temperature sensitivity of fiber optic polarimetric sensors by selecting a PM fiber type with appropriate birefringence and length [74,75].

**Figure 13:**
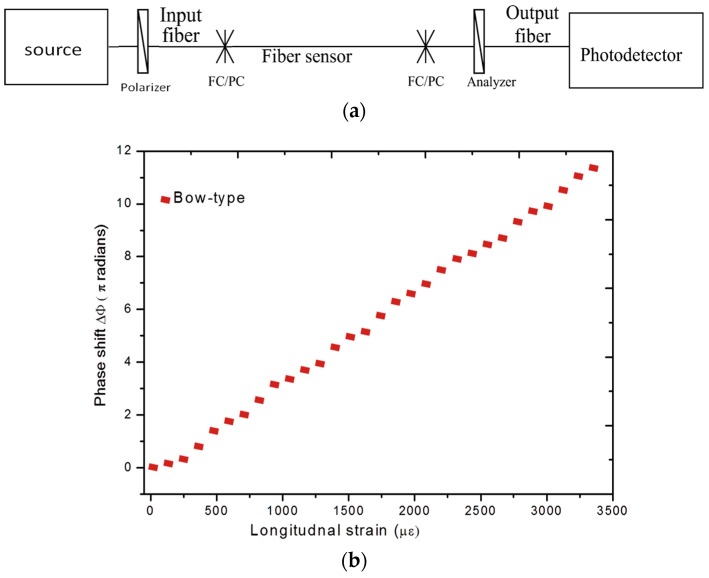
(**a**) Experimental setup for measurements with fiber optic polarimetric sensors in intensity domain [66]; and (**b**) Change of the polarization of fiber optic polarimetric sensors as a function of strain applied to a composite sample [76].

For fiber optic polarimetric sensors the phase difference between the two orthogonal polarizations, can be extracted using an experimental setup consisting of a tunable laser source and a polarimeter/polarization control system [76]. Fiber optic polarimetric sensors can be also operated in intensity domain with the help of a polarizer-analyzer arrangement and the experimental setup for operation in intensity domain is shown in Figure 13a. For polarimetric sensors the change in the output intensity at a wavelength λ due to externally applied longitudinal strain can be described by the formula [73]:

(8) $$I_{s}(\lambda )=\frac{I_{0}}{2}\left[1+\cos(\Delta \Phi )\right]$$

where, *I*_0_ is the input initial intensity.

For such polarimetric sensors operating in the intensity domain periodic variations in the output intensity can be associated with applied strain or temperature [77]. It is reported that low strain sensitive polarimetric sensors such as HB-PM-PCF polarimetric sensor guarantee a linear response for a wide range of applied strain values [77]. The change of the polarization of fiber optic polarimetric sensors based on bow tie type fiber as a function of strain applied to a composite sample is as shown in Figure 13b. Moreover, the insignificant temperature sensitivity of PM-HB-PM-PCFs make them the most appropriate candidates for composite strain measurements [77,78]. However, for local strain/temperature measurements of large composite structures polarimetric sensors cannot be employed as the polarimetric sensors measures average strain over the length of the sensing fiber.

### 5.4. Fiber Optic Micro Bend Sensors

**Figure 14:**
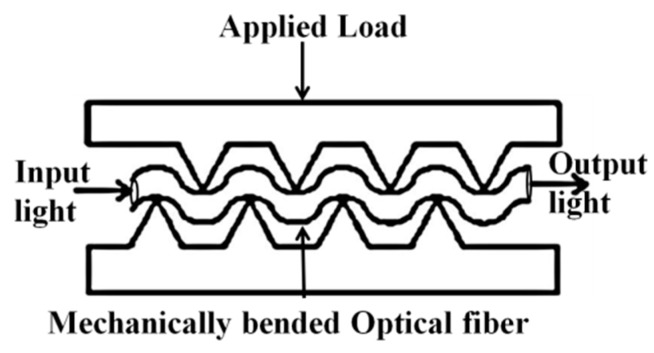
Micro bend sensor concept [40].

Fiber optic micro bend sensors are capable of measuring parameters such as pressure, acceleration, displacement, temperature and strain [40,79,80]. Microbending is typically caused by defects and small geometrical perturbations in the optical fiber. The curvature of radius of microbends is in the order of micrometers. Microbends cause coupling between propagating and radiation modes [40] which results in the loss of intensity of the light propagating through the fiber. Figure 14 shows the concept underpinning a typical microbending sensor. Fiber optic micro bend sensor is typically formed by passing the fiber between two sets of corrugations as shown in Figure 14.

**Figure 15:**
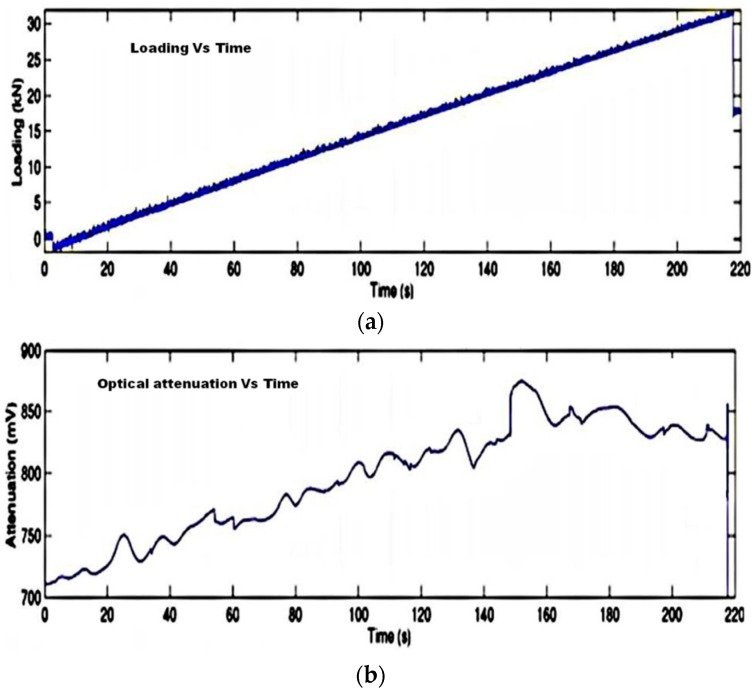
The temporal profiles corresponding to loading (**a**) and optical signal attenuation (**b**) [40].

Delamination or cracking in the layers of a composite produces corrugations in that cause microbending in an embedded optical fiber. Specifically during delamination of a multilaminated composite structure the stress fields in a damaged layer and an undamaged layer are different. The higher stress level in the damaged layer causes the optical fiber to micro-bend in the material resulting in the attenuation of the output, and therefore this method can be used to measure the strain field due to delamination in a composite material [40]. As an example the temporal profile of loading in the composite material and the corresponding optical signal attenuation are shown in Figure 15. Another method of damage detection using the fiber optic micro bend sensor utilizes mechanical/acoustic waves propagating in the composite material [80,81]. When a mechanical wave hits an optical fiber, it bends the fiber locally and so some coupling between propagating and radiation modes may appear. As a result, temporal features in the measured optical signal can be related to stress waves released by delamination, matrix cracking or reinforcing fibre rupture [40]. The main disadvantage of micro bend sensors is their low accuracy which makes them unsuitable for precise stress measurements.

### 5.5. Distributed Fiber Optic Sensors

Distributed fiber-optic sensors (DFS) are capable of providing a continuous measurand profile over the length of the optical fiber and thus are very promising for strain/temperature measurements in large structures such as bridges, buildings and pipelines [41,82,83]. However given the nature of composite structures, the length is normally limited to 80 m or less with a strain or temperature requirement of at least 1 °C or 20 µε with a few centimeters resolution. DFSs are categorized into several types based on the sensing technology employed and the related physical effect underpinning the operating principle: (i) optical time–domain reflectometry (OTDR) and optical frequency–domain reflectometry (OFDR), both based on Rayleigh scattering; (ii) Raman optical time–domain reflectometry (ROTDR) and Raman optical frequency–domain reflectometry (ROFDR), both based on Raman scattering; and (iii) Brillouin optical time–domain reflectometry (BOTDR) and Brillouin optical frequency-domain reflectometry (BOFDR), both based on Brillouin scattering [84,85,86].

**Figure 16:**
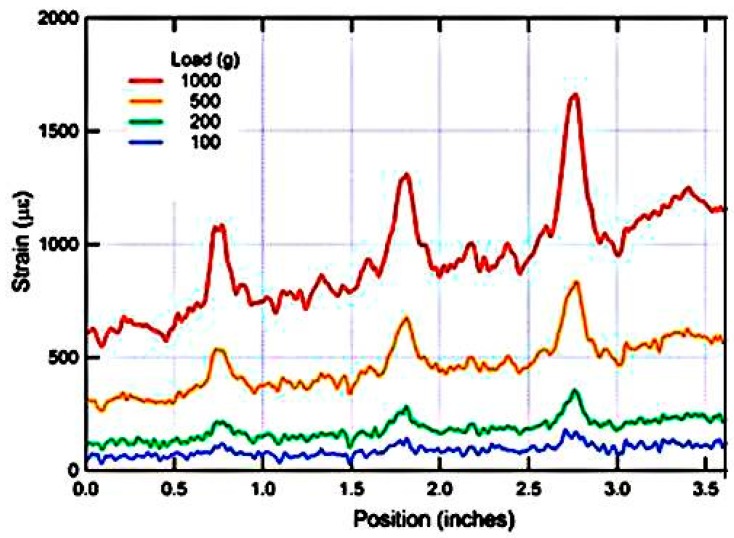
Strain measured by the optical fiber bonded to a composite sample for various loading conditions (data redrawn from *Luna Engineering Note EN-FY1317*).

OTDR and OFDR are the first generation of fiber optic distributed sensors, based on the use of Rayleigh scattering to reflect the attenuation profiles of long-range optical fiber links [83,84]. An example of strain measurement by OFDR technique for various loading conditions is shown in Figure 16. 

An optical pulse is launched into an optical fiber and the power of the Rayleigh backscattered light is detected using a photo detector as the light pulse propagates along the fiber link [85]. Frequency based Brillouin method can provide rapid strain/temperature sensing [87,88]. BOFDR can be employed to measure strain/temperature variations as small as 1 °C or 20 µε with few centimeters of resolution [88,89,90]. For BOFDR based long distance measurements single-mode fibers are used [89,91]. ROTDR is an appropriate candidate for temperature measurements in composites since the intensity ratio between anti-Stokes components and Stokes components of the ROTDR response provide information about temperature [88]. ROTDR can be employed to measure temperature variations as small as 0.1 °C with a few meters of resolution [87,88]. Multimode fibers are commonly used for ROTDR based short distance measurements [89]. In the case of composite strain/temperature measurements from the above mentioned categories of DFS, an appropriate sensing technology can be selected based on the application and its requirements, specifically BOFDR is more suitable for strain measurements and ROTDR is more suitable for temperature measurements in composite material [88,89,91]. Other than the technologies mentioned above, quasi-distributed optical fiber sensors can be realized by multiplexing a number of grating-based sensors [85].

### 5.6. Hybrid Sensing Approaches for Simultaneous Strain and Temperature Measurements in Composite Materials

In most of practical applications of composite materials, the key requirement is to measure more than one parameter. For example, for SHM applications in composite parts such as helicopter rotor blades, wind turbines and aircraft structures a simultaneous monitoring of temperature and strain is favorable. Several FOSs such as FBGs, PM polarimetric sensors, etc are capable of measuring strain and temperature, but there is always an issue of accurate discrimination between these two influencing factors [35,37]. One of the simplest and promising methods for simultaneous measurement of strain and temperature in composite parts is the hybrid fiber optic sensing approach; in this two or more FOS operate in a combined manner to eliminate the disadvantages of individual FOSs providing accurate and independent strain/temperature information [92]. Many researchers have investigated different hybrid sensing approaches for simultaneous strain and temperature measurements such as using a combination of FBG sensors with various type sensors such as long period gratings (LPG) [55,92], Fabry-Perot interferometer sensors [54], PCF modal interferometers [93], and fiber loop mirrors using a small core micro-structured fiber [94]. Sensors based on gratings written in micro structured fibers and standard optical fibers have also been reported for discrimination between strain and temperature [95]. Previously we reported a hybrid approach which involves an FBG sensor and a HB-PM-PCF polarimetric sensor for simultaneous measurement of strain and temperature [96] of composite materials. In the context of composite structures, the temperature sensitivity of a FOS can be influenced by the thermal strain of the composite material [16]. Sensors based on stripped HB-PM-PCF based polarimetric fibers have been reported for thermal strain and thermal expansion measurements in composite materials [15]. A hybrid optical fiber sensor embedded in a composite material which is capable of discriminating between strain, temperature and thermal strain has great potential for future FOS based SHM applications.

In the previous sections we have detailed the major types of FOS used for strain and temperature measurements in composite materials. Appropriate sensor selection for any application is only possible by identifying the particular features of all FOS. The FOS types discussed in this review are compared in Table 1.

## 6. Recent Trends, Issues and Future Challenges of the FOS Technology

Embedding FOS inside composite materials is a minimally invasive technique; but for industrial applications still there exist a few issues which are under investigation and in this section we discuss those challenging issues.

**Figure 17:**
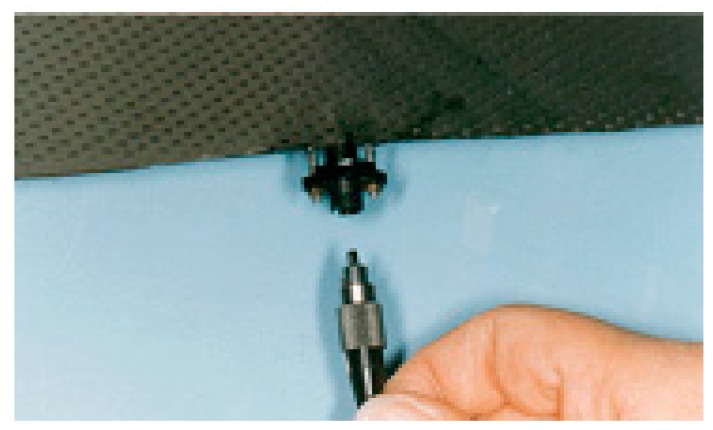
Egress optical fiber with connector.

One of the challenges with composite structures embedded with FOS is the provision of a reliable method for connecting to the sensor [97,98]. Custom designed connectors with ingress/egress optical fiber ends are promising technique, and one example of such a connector is shown in Figure 17. However, such egress optical fibers with connectors may cause the composite to become brittle at the edges of the structure [99]. Also edge trimming of the composite after the connector installation is impossible. Free space coupling is more promising than surface and edge mounted connectors [100]. For example, in [101] a novel method of free-space passive coupling of light into FBG sensors is reported which consists of an angled 45° mirror integrated directly into the fiber. Also to resolve the connection issues associated with ingress/egress optical fiber ends of L-shaped composite a reconnection technique is established by situating the optical fiber connector on a 6-axis automatic stage together with the assistance of CCD camera [102].

**Table 1:** Comparison of FOSs.

FOS Technology	Advantages	Disadvantages	Remarks	Main Applications
Standard FBGs	Most accepted technology, allows for point measurements of strain and temperature	Temperature and strain cross sensitivity issues	Typical strain sensitivity ~1.2 pm/µε and typical temperature sensitivity ~11.6 pm/°C	Strain, temperature, vibration, cure process, localized damage, *etc.*
FBGs written in MOF	Can discriminate both axial and transverse strain components of composite material with insignificant temperature sensitivity	FBGs written in bow-tie fibers have temperature and strain cross sensitivity. But FBGs written in MOF have lower strain sensitivity compared to FBGs written in bow-tie fibers.	The cross-sensitivity issue can be resolved by using FBGs written in low temperature sensitive MOFs	Multi directional strain sensing, localized damage, *etc.*
Interferometric fiber optic sensors	Possesses higher temperature and strain sensitivities and are flexible in terms of size	Temperature and strain cross sensitivity issue, and brittle sensor	The cross-sensitivity issue can be resolved by using low temperature sensitive MOFs	Strain, temperature, vibration, cure process, localized damage, *etc.*
Polarimetric sensors	Sensitivity can be tuned by choosing different optical fiber types and sensor lengths	Difficult to measure strain/temperature at localized points, provide information averaged over the sensor’s length	The cross-sensitivity issue can be resolved by using low temperature sensitive HB-PM-PCF	Strain, temperature, vibration, cure process, *etc.*
Fiber optic micro bend sensors	Can measure continuous strain profile in a composite material using single optical fiber	Low accuracy	Output signal is strongly attenuated by any mechanical wave propagating in the composite material	Delamination and damage detection
Distributed sensors	Can measure continuous strain/temperature profile in a composite material using single optical fiber	For better resolution require the use of spectral demodulation techniques that are expensive and bulky	Appropriate sensing technology can be selected based on the application and its requirements	Strain, temperature, delamination, damage detection
Hybrid sensors	Two or more FOS operate in a combined manner to eliminate the disadvantages of individual FOSs providing accurate and independent strain/temperature information	Since two or more sensors are employed complicated interrogation methods are needed	Capable of discriminating between strain, temperature and thermal strain	Strain, thermal strain, temperature, vibration, cure process, damage point, *etc.*

The second issue is the structural damage to the composite material due to the fact that optical fibers have larger diameter compared to reinforcement fibers. As a solution researchers are considering new optical fibers with a reduced diameter and an optimized coating. One example of such fibers is a thinner optical fiber with 80 micron diameter known as Draw Tower Grating (DTG) fiber, which should be less invasive when embedded within a laminate composite structure [103].

Another issue related to embedding of FOS in a composite part in weight or space sensitive applications is the large size of conventional FOS interrogators which could present difficulties for sensing of composite parts that for example are in constant motion, such as helicopter rotor blades and wind turbine structures [104]. To an extent this issue can be resolved by adopting miniaturized interrogation modules based on photonic integrated circuits (PIC) or flexible polymer waveguides [105,106]. Such surface attachable flexible interrogators allow for integration of photo detector arrays with wireless communication technology and thus have strong potential in smart sensing of composite parts in motion.

**Figure 18:**
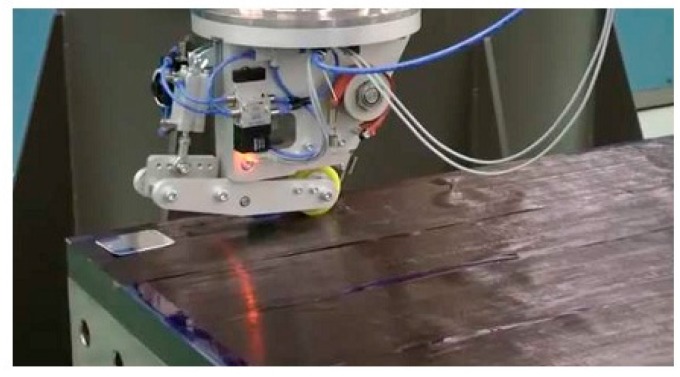
Automated optical fiber placement system (from “*Airborne: the future in composites*” website).

Finally, it must be recognised the FOS embedding procedure is often a labor intensive task. Therefore, ideally it requires a reliable automated optical fiber placement system that matches well with the existing industrial composite production processes. Several composite manufactures, such as for example Airborne, have introduced automated fiber placement system that provide control over embedding depth, pre-strain, position and alignment. Such an all automated fiber placement system is shown in Figure 18. The biggest challenge in using most of automated FOS embedding systems, is that it is difficult to ensure an adequate repeatability of the FOS placement. In addition, during such an automated fabrication process the safety of delicate areas of FOS (such as grating written area of FBG, spliced and buffer coating removed areas of sensing optical fiber, *etc.*) and control over the alignment of specialized optical fibers such as micro structured optical fibers are not well assured. Thus, an automated fiber placement system together with an X-ray based micro controlled tomography could be a suitable solution for quality control of the embedded FOS and a realistic option for appropriate alignment of optical fibers for future smart sensing applications.

## 7. Conclusions

This paper presents a comprehensive overview of the FOS sensors used for strain/temperature sensing of composite materials. This review article provides essential information regarding many aspects, including different type composite materials, properties, and their performance, the relevance of fiber optic sensor (FOS) for composite material sensing applications, future challenges of embedded FOS and recent trends. Embedded FOS have proved themselves useful to smart sensing applications of composite materials in wide range of areas that include aerospace, structural, civil and the sports industry. Extensive research and development continues worldwide and will inevitably make this technology more commercially viable and more beneficial to society in general.

## References

[1] Garg D.P., Zikry M.A., Anderson G.L., Gros X.E. (2001). Current and potential future research activities in adaptive structures: An ARO perspective. Smart Mater. Struct..

[2] Balageas D. (2006). Introduction to structural health monitoring. Structural Health Monitoring.

[3] Cai J., Qiu L., Yuan S., Shi L., Liu P., Liang D. (2012). Structural Health Monitoring for Composite Materials. Composites and Their Applications.

[4] Zhu Y.K., Gui Y.T., Rong S.L., Hong Z. (2011). A review of optical NDT technologies. Sensors.

[5] Gros X.E. (2000). Current and Future Trends in Non-Destructive testing of Composite Materials. Ann. Chim. Sci. Mater..

[6] Ye X.W., Su Y.H., Han J.P. (2014). Structural health monitoring of civil infrastructure using optical fiber sensing technology: A comprehensive review. The Sci. World J..

[7] Méndez A., Csipkes A. (2013). Overview of fiber optic sensors for NDT applications. Nondestructive Testing of Materials and Structures.

[8] Chung D.D.L. (2010). Composite Materials: Science and Applications.

[9] Hull D., Clyne T.W. (1996). An Introduction to Composite Materials.

[10] Kaw A.K. (2005). Mechanics of Composite Materials.

[11] Bunsell A.R., Jacques R. (2005). Fundamentals of Fibre Reinforced Composite Materials.

[12] Fernando G. Sensors and Composites: Requirements, Opportunities and Complexity. Presented at the 1st International Workshop on Embedded Optical Sensors for Composite Materials.

[13] Kaufmann M. Composite Materials: State-of-the-art and Future Challenges. Presented at the 1st International Workshop on Embedded Optical Sensors for Composite Materials.

[14] Elmar W., Bernhard J. Composites Market Report 2014. http://www.eucia.eu/userfiles/files/20141008_market_report_grpcrp.pdf.

[15] Ramakrishnan M., Rajan G., Semenova Y., Boczkowska A., Domański A., Wolinski T., Farrell G. (2013). Measurement of thermal elongation induced strain of a composite material using a polarization maintaining photonic crystal fiber sensor. Sens. Actuators A Phys..

[16] Ramakrishnan M., Rajan G., Semenova Y., Lesiak P., Domanski A., Wolinski T., Boczkowska A., Farrell G. (2011). The influence of thermal expansion of a composite material on embedded polarimetric sensors. Smart Mater. Struct..

[17] Lesiak P., Szeląg M., Budaszewski D., Plaga R., Mileńko K., Rajan G., Semenova Y., Farrell G., Boczkowska A., Domański A. (2012). Influence of lamination process on optical fiber sensors embedded in composite material. Measurement.

[18] Degrieck J., Wim D.W., Patricia V. (2001). Monitoring of fibre reinforced composites with embedded optical fibre Bragg sensors, with application to filament wound pressure vessels. NDT&E Int..

[19] Hernández-Moreno H., Collombet F., Douchin B., Choqueuse D., Davies P., Velázquez J.G. (2009). Entire life time monitoring of filament wound composite cylinders using Bragg grating sensors: I. Adapted tooling and instrumented specimen. Appl. Compos. Mater..

[20] Keulen C., Rocha B., Yildiz M., Suleman A. (2011). Embedded fiber optic sensors for monitoring processing, quality and structural health of resin transfer molded components. J. Phys. Conf. Ser..

[21] Bektaş G., Boz T., Keulen C.J., Yıldız M., Öztürk C., Menceloğlu Y.Z., Suleman A. Fiber Bragg grating and etched optic sensors for flow and cure monitoring of resin transfer molded composite structures. Proceedings of the 18th International Conference on Composite Materials (ICCM18).

[22] Tuccillo F., Antonucci V., Calabro A.M., Giordano M., Nicolais L. (2005). Practical and theoretic analysis of resin flow in vacuum assisted resin transfer molding processes. Macromolecular Symposia.

[23] Yuan S., Huang R., Rao Y. (2004). Internal strain measurement in 3D braided composites using co-braided optical fiber sensors. J. Mater. Sci. Technol..

[24] Antonucci V., Esposito M., Ricciardi M.R., Raffone M., Zarrelli M., Giordano M. (2011). Permeability characterization of stitched carbon fiber preforms by fiber optic sensors. Express Polym. Lett..

[25] Jensen D.W., Pascual J. (1990). Degradation of graphite/bismaleimide laminates with multiple embedded fiber-optic sensors. Proc. SPIE.

[26] Lee D.C., Lee J.J., Yun S.J. (1995). The mechanical characteristics of smart composite structures with embedded optical fiber sensors. Compos. Struct..

[27] Güemes J., Perez J.S. (2013). Fiber Optics Sensors. New Trends in Structural Health Monitoring.

[28] Luycks G., Voet E., Lammens N., Degrieck J. (2011). Strain Measurements of Composite Laminates with Embedded Fibre Bragg Gratings: Criticism and Opportunities for Research. Sensors.

[29] Błażejewski W., Gąsior P., Kaleta J. (2011). Application of Optical Fiber Sensors to Measuring the Mechanical Properties of Composite Materials and Structures. Advances in Composite Materials. Ecodesign and Analysis.

[30] Emmanwori L., Shivakumar K.N. Structural performance of composite laminates with embedded fiber optic sensor under tension and compression loads. Proceedings of the 43rd Annual Conference of American Institute of Aeronautics and Astronautics.

[31] Ciang C.C., Jung R.L., Hyung J.B. (2008). Structural health monitoring for a wind turbine system: A review of damage detection methods. Meas. Sci. Technol..

[32] Zhou G., Sim L.M. (2002). Damage detection and assessment in fibre-reinforced composite structures with embedded fibre optic sensors-review. Smart Mater. Struct..

[33] Di Sante R. (2015). Fibre Optic Sensors for Structural Health Monitoring of Aircraft Composite Structures: Recent Advances and Applications. Sensors.

[34] Minakuchi S. From Material Characterization to Product Quality Control: Applicability of Fibre-Optic Sensors to Composites Process Monitoring. Presented at the 1st International Workshop on Embedded Optical Sensors for Composite Materials.

[35] Du W., Tao X.M., Tam H.Y., Choy C.L. (1998). Fundamentals and applications of optical fiber Bragg grating sensors to textile structural composites. Compos. Struct..

[36] Di Sante R., Donati L., Troiani E., Proli P. (2014). Reliability and accuracy of embedded fiber Bragg grating sensors for strain monitoring in advanced composite structures. Met. Mater. Int..

[37] Lau K.T. (2014). Structural health monitoring for smart composites using embedded FBG sensor technology. Mater. Sci. Technol..

[38] Rao Y.J. (2006). Recent progress in fiber-optic extrinsic Fabry-Perot interferometric sensors. Opt. Fiber Technol..

[39] Murukeshan V.M., Chan P.Y., Ong L.S., Asundi. A. (1999). On-line health monitoring of smart composite structures using fiber polarimetric sensor. Smart Mater. Struct..

[40] Rippert L., Jean-Michel P., Martine W., Sabine V.H. (2002). Fibre optic sensor for continuous health monitoring in CFRP composite materials. Proc. SPIE.

[41] Murayama H., Kazuro K., Hiroshi N., Akiyoshi S., Kiyoshi U. (2003). Application of fiber-optic distributed sensors to health monitoring for full-scale composite structures. J. Intell. Mater. Syst. Struct..

[42] López-Higuera J.M., Rodriguez Cobo L., Quintela Incera A., Cobo A. (2011). Fiber optic sensors in structural health monitoring. J. Lightwave Technol..

[43] Gerard F.F. Fibre optic sensor systems for monitoring composite structures. Porceedings of the RP Asia 2005 Conference.

[44] Montanini R., d’Acquisto L. (2007). Simultaneous measurement of temperature and strain in glass fiber/epoxy composites by embedded fiber optic sensors: I. Cure monitoring. Smart Mater. Struct..

[45] Glisic B., Inaudi D. (2007). Fibre Optic Methods for Structural Health Monitoring.

[46] Hill K.O., Meltz G. (1997). Fiber Bragg grating technology fundamentals and overview. J. Lightwave Technol..

[47] Raman K. (1999). Fiber Bragg Gratings.

[48] Yashiro S., Okabe T., Toyama N., Takeda N. (2007). Monitoring damage in holed laminates using embedded chirped FBG sensors. Int. J. Solids Struct..

[49] Dong X., Zhang H., Liu B., Miao Y. (2011). Tilted fiber Bragg gratings: Principle and sensing applications. Photonic Sens..

[50] Zhao Y., Yanbiao L. (2004). Discrimination methods and demodulation techniques for fiber Bragg grating sensors. Opt. Lasers Eng..

[51] Xu M.G., Archambault J.L., Reekie L., Dakin J.P. (1994). Discrimination between strain and temperature effects using dual-wavelength fibre grating sensors. Electron. Lett..

[52] James S.W., Dockney M.L., Tatam R.P. (1996). Simultaneous independent temperature and strain measurement using in-fibre Bragg grating sensors. Electron. Lett..

[53] Samer K.B., Sun T., Grattan K.T.V. (2008). Simultaneous measurement of temperature and strain with long period grating pairs using low resolution detection. Sens. Actuators.

[54] Oliveira R.D., Ramos C.A., Marques A.T. (2008). Health monitoring of composite structures by embedded FBG and interferometric Fabry-Perot sensors. Comput. Struct..

[55] Guan B.O., Tam H.Y., Tao X.M., Dong X.Y. (2000). Simultaneous strain and temperature measurement using a superstructure fiber Bragg grating. IEEE Photonics Technol. Lett..

[56] Rajan G., Ramakrishnan M., Lesiak P., Semenova Y., Wolinski T., Boczkowska A., Farrell G. (2012). Composite materials with embedded photonic crystal fiber interferometric sensors. Sens. Actuators A Phys..

[57] Kreuzer M. (2006). Strain Measurement with Fiber Bragg Grating Sensors.

[58] Sonnenfeld C., Sulejmani S., Geernaert T., Eve S., Lammens N., Luyckx G., Eli V., Thienpont H. (2011). Microstructured optical fiber sensors embedded in a laminate composite for smart material applications. Sensors.

[59] Guemes J.A., Menendez J.M. (2002). Response of Bragg grating fiber-optic sensors when embedded in composite laminates. Compos. Sci. Technol..

[60] Luyckx G., Voet E., Geernaert T., Chah K., Nasilowski T., De Waele W., van Paepegem W., Becker M., Bartelt H., Urbanczyk W. (2009). Response of FBGs in microstructured and bow tie fibers embedded in laminated composite. IEEE Photonics Technol. Lett..

[61] Azmi A.I., Deep S., Wenjuan S., John C., Gang D.P. (2011). Performance enhancement of vibration sensing employing multiple phase-shifted fiber Bragg grating. J. Lightwave Technol..

[62] Webb D.J. (2012). Polymer optical fibre Bragg gratings. OSA Tech. Dig..

[63] Kalli K., Dobb H.L., Webb D.J., Carroll K., Themistos C., Komodromos M., Peng G.-D., Fang Q., Boyd I.W. (2007). Development of an electrically tuneable Bragg grating filter in polymer optical fibre operating at 1.55 µm. Meas. Sci. Technol..

[64] Zhang W., Webb D.J., Peng G.-D. (2012). Investigation in to time response of polymer fibre Bragg grating based humidity sensors. J. Lightwave Technol..

[65] Rajan G., Ramakrishnan M., Semeonva Y., Ambikairajah E., Farrell G., Peng G.D. (2014). Experimental study and analysis of a polymer fibre Bragg grating embedded in a composite material. J. Lightwave Technol..

[66] Lee B.H., Kim Y.H., Park K.S., Eom J.B., Kim M.J., Rho B.S., Choi H.Y. (2012). Interferometric fiber optic sensors. Sensors.

[67] Udd E., Spillman W.B. (2011). Fiber Optic Sensors: An Introduction for Engineers and Scientists.

[68] Liu T., Wu M., Rao Y., Jackson D.A., Fernando G.F. (1998). A multiplexed optical fibre-based extrinsic Fabry-Perot sensor system for *in-situ* strain monitoring in composites. Smart Mater. Struct..

[69] Leng J.S., Asundi A. (2003). Structural health monitoring of smart composite materials by using EFPI and FBG sensors. Sens. Actuators A Phys..

[70] Fernando G.F., Liu T., Crosby P., Doyle C.A., Martin D.B., Ralph B., Badcock R. (1997). A multi-purpose optical fibre sensor design for fibre reinforced composite materials. Meas. Sci. Technol..

[71] Woliński T.R. (2000). I Polarimetric optical fibers and sensors. Prog. Opt..

[72] Domanski A.W., Tomasz R.W., Wojtek J.B. (1994). Polarimetric fiber optic sensors: State of the art and future. Proc. SPIE.

[73] Woliński T.R., Lesiak P., Domański A.W. (2008). Polarimetric optical fiber sensors of a new generation for industrial applications. Tech. Sci..

[74] Ma J., Asundi A. (2001). Structural health monitoring using a fiber optic polarimetric sensor and a fiber optic curvature sensor-static and dynamic test. Smart Mater. Struct..

[75] Hogg W.D., Roderick D.T. (1990). Polarimetric fibre optic structural strain sensor characterisation. Proc. SPIE.

[76] Domański A.W., Lesiak P., Karolina M., Boczkowska A., Budaszewski D., Ertman S., Woliński T.R. (2009). Temperature-insensitive fiber optic deformation sensor embedded in composite material. Photonics Lett. Pol..

[77] Kim D.H., Kang J.U. (2007). Analysis of temperature-dependent birefringence of a polarization-maintaining photonic crystal fiber. Opt. Eng..

[78] Murukeshan V.M., Chan P.Y., Ong L.S., Asundi A. (2000). Effects of different parameters on the performance of a fiber polarimetric sensor for smart structure applications. Sens. Actuators A Phys..

[79] Berthold J.W. (1995). Historical review of microbend fiber-optic sensors. J. Lightwave Technol..

[80] Pandey N.K., Yadav B.C. (2006). Embedded fibre optic microbend sensor for measurement of high pressure and crack detection. Sens. Actuators A Phys..

[81] Wevers M., Rippert L., Papy J.M., van Huffel S. (2006). Processing of transient signals from damage in composite materials monitored with embedded intensity-modulated fiber optic sensors. NDT&E Int..

[82] Adachi S. Distributed optical fiber sensors and their applications. Proceedings of the SICE Annual Conference.

[83] Niklès M. (2007). Fibre optic distributed scattering sensing system: Perspectives and challenges for high performance applications. Proc. SPIE.

[84] Grattan K.T.V., Sun T. (2000). Fiber optic sensor technology: An overview. Sens. Actuators A Phys..

[85] Kersey A.D. (1996). A review of recent developments in fiber optic sensor technology. Opt. Fiber Technol..

[86] Bao X., Liang C. (2011). Recent progress in Brillouin scattering based fiber sensors. Sensors.

[87] Hartog A.H. (2002). Progress in Distributed Fiber Optic Temperature Sensing. Environmental and Industrial Sensing. Proc. SPIE.

[88] Culshaw B. (2004). Optical fiber sensor technologies: Opportunities and-perhaps-pitfalls. J. Lightwave Technol..

[89] Ravet F. (2011). Distributed Brillouin Sensor Application to Structural Failure Detection. New Developments in Sensing Technology for Structural Health Monitoring.

[90] Bernini R., Minardo A., Zeni L. (2012). Distributed Sensing at centimetre-scale spatial resolution by BOFDA: Measurements and signal processing. IEEE Photonics J..

[91] Zeng X., Bao X., Chia Y.C., Theodore W.B., Anthony W.B., Michael D.D., Graham F., Alexander L.K., Anastasis V.G. (2002). Strain measurement in a concrete beam by use of the Brillouin-scattering-based distributed fiber sensor with single-mode fibers embedded in glass fiber reinforced polymer rods and bonded to steel reinforcing bars. Appl. Opt..

[92] Patrick H.J., Williams G.M., Kersey A.D., Pedrazzani J.R. (1996). Hybrid fibre Bragg grating/long period grating sensor for strain/temperature discrimination. IEEE Photonics Technol. Lett..

[93] Dong B., Hao J., Liaw C., Lin B., Tjin S.C. (2010). Simultaneous strain and temperature measurement using a compact photonic crystal fiber inter-modal interferometer and a fiber Bragg grating. Appl. Opt..

[94] Andre R.M., Marques M.B., Roy P., Frazao O. (2010). Fiber Loop Mirror Using a Small Core Micro-structured Fiber for Strain and Temperature Discrimination. IEEE Photonics Technol. Lett..

[95] Frazão O., Carvalho J.P., Ferreira L.A., Araújo F.M., Santos J.L. (2005). Discrimination of strain and temperature using Bragg gratings in microstructured and standard optical fibres. Meas. Sci. Technol..

[96] Rajan G., Ramakrishnan M., Semenova Y., Karolina M., Lesiak P., Domanski A.W., Wolinski T.R., Farrell G. (2012). A Photonic Crystal Fiber and Fiber Bragg Grating-Based Hybrid Fiber-Optic Sensor System. Sens. J..

[97] Kang H.K., Park J.W., Ryu C.Y., Hong C.S., Kim C.G. (2000). Development of fibre optic ingress/egress methods for smart composite structures. Smart Mater. Struct..

[98] Green A.K., Zaidman M., Shafir E., Tur M., Gali S. (2000). Infrastructure development for incorporating fibre-optic sensors in composite materials. Smart Mater. Struct..

[99] Simon W.R., William R.P. (2001). Apparatus for Ingress and Egress of Fiber Optic Sensor Leads from the Surface of Composite Parts and a Method for the Manufacture Thereof. U.S. Patent.

[100] Kinet D., Mégret P., Goossen K.W., Qiu L., Heider D., Caucheteur C. (2014). Fiber Bragg Grating Sensors toward Structural Health Monitoring in Composite Materials: Challenges and Solutions. Sensors.

[101] Qiu L., Goossen K.W., Heider D., Wetzel E.D. (2010). Nonpigtail optical coupling to embedded fiber Bragg grating sensors. Opt. Eng..

[102] Minakuchi S., Umehara T., Takagaki K., Ito Y., Takeda N. (2013). Life cycle monitoring and advanced quality assurance of L-shaped composite corner part using embedded fiber-optic sensor. Compos. Part A Appl. Sci. Manuf..

[103] Chojetzki C., Rothhardt M., Ommer J., Unger S., Schuster K., Mueller H.R. (2005). High-reflectivity draw-tower fiber Bragg gratings—Arrays and single gratings of type II. Opt. Eng..

[104] Palmer D.D., Engelbart R.W., Vaccaro C.M. (2004). Future Directions Relative to NDE of Composite Structures. SAE Tech. Pap..

[105] Van H., Bram G.L., Erwin B., Jeroen M., Sandeep K., Oliver M., David J.W., Kate S., Peter V.D., Geert V.S. (2012). Ultra small integrated optical fiber sensing system. Sensors.

[106] Missinne J., Geert V.S., Bram V.H., Kristof V.C., Tim V.G., Peter D., Jan V., Peter V.D. (2009). An array waveguide sensor for artificial optical skins. Proc. SPIE.

